# Congenital Absence of Left Circumflex Artery: A Case Report and Review of the Literature

**DOI:** 10.1155/2017/6579847

**Published:** 2017-10-30

**Authors:** Setri Fugar, Lydia Issac, Alexis Kofi Okoh, Christelle Chedrawy, Nadia El Hangouche, Neha Yadav

**Affiliations:** ^1^Department of Internal Medicine, John H. Stroger Hospital of Cook County, 1901 West Harrison Street, Chicago, IL 60612, USA; ^2^Department of Cardiothoracic and Vascular Surgery, Barnabas Heart Hospitals, West Orange, NJ 07052, USA; ^3^Department of Radiology, John H. Stroger Hospital of Cook County, 1901 West Harrison Street, Chicago, IL 60612, USA; ^4^Department of Cardiology, John H. Stroger Hospital of Cook County, 1901 West Harrison Street, Chicago, IL 60612, USA

## Abstract

Congenital absence of the left circumflex artery is a rare coronary anomaly with few reported cases in the literature. These patients are usually diagnosed incidentally when they undergo coronary angiography or coronary CT to rule out underlying coronary artery disease. In this article, we report a case of a 46-year-old man who was incidentally found to have a congenitally absent left circumflex artery with a superdominant right coronary artery after a workup was initiated for frequent premature ventricular contractions and regional wall motion on echocardiogram. A review of the clinical presentation, symptoms, and diagnostic modalities used to diagnose this entity is presented.

## 1. Introduction

Congenital absence of the left circumflex (LCX) artery is a rare coronary anomaly with an incidence of 10 in 1495 (0.0067%) patients [1, 2]. Due to its uncommon nature, diagnosis can be challenging, especially among patients who present with acute cardiac symptoms. It may initially be misdiagnosed as complete occlusion of the left circumflex artery [3]. A better understanding of the natural history and clinical implications of this condition may allow us to diagnose and manage this condition with an algorithmic approach. We present a case of congenitally absent left circumflex artery with a superdominant right coronary artery (RCA) detected by coronary angiography and confirmed by coronary CT. Also, a review of previously published cases in literature and their clinical outcomes is presented.

## 2. Methods

**Figure 1:**
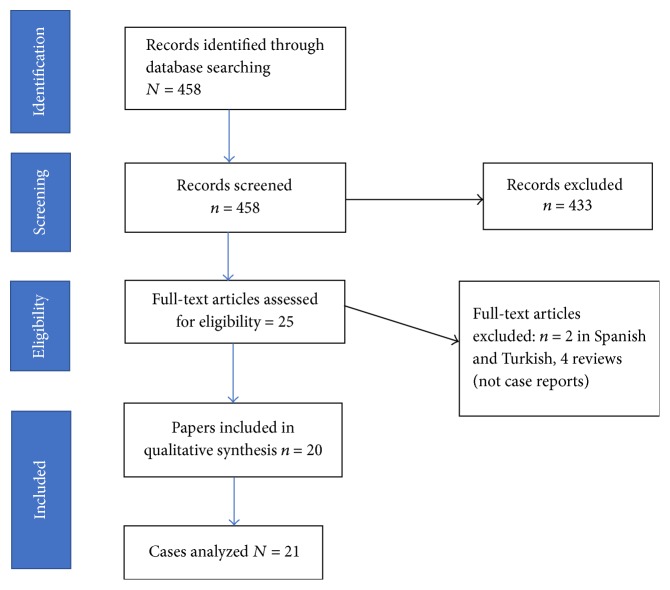
PRISMA flowchart: data collection and selection of cases.

In addition to the present case, a systematic review of case reports/short cases in OVID looking at patients who presented with congenitally absent left circumflex artery was performed. The review only considered papers published in the English language. An electronic search strategy with the keywords Left circumflex AND Absent OR Absence AND Congenital was used. Duration of published papers was defined between and 1946 and October 2016. Definitions used for case-inclusion were (a) age greater than 18 years and (b) diagnosis of left circumflex artery abnormality either by CT/MRI or coronary angiography (Figure 1).

## 3. Case Presentation

A 46-year-old man with no significant medical history presented to the emergency department with an episode of transient loss of consciousness after a mechanical fall. He had no preceding chest pain or palpitations. There was no history of syncope, presyncope, loss of consciousness, diabetes, hypertension, or seizure disorder. On admission, vital signs showed heart rate of 88 bpm, BP of 115/83 mmhg, oxygen saturation of 95% on room air, and respiratory rate of 18 cpm. Physical examination was within normal limits including no neurological deficits. Computed tomography of the brain showed normal findings with no evidence of intracerebral hemorrhage. Labs were all within normal limits including a negative troponin.

**Figure 2:**
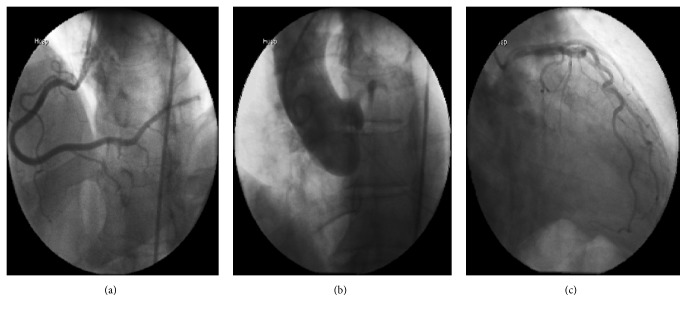
Coronary angiogram: (a) LAO-CAU view of superdominant RCA, (b) aortic root shot to rule out anomalous origin of the LCX, and (c) RAO caudal view showing absent left circumflex in the AV groove.

**Figure 3:**
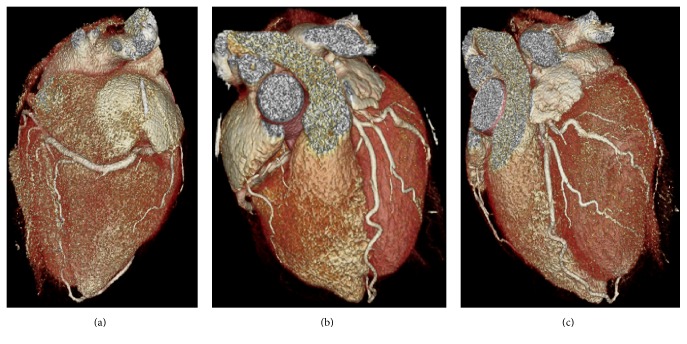
Coronary CT views: (a) left ventricle being supplied by branches of the RCA, (b) LAD in the interventricular groove, and (c) absent left circumflex artery.

While the patient was being monitored in the emergency department he was noted to be in sinus rhythm with frequent premature ventricular contractions (PVCs). An EKG showed sinus rhythm with T wave inversions in the inferior leads and frequent PVCs. A transthoracic echocardiogram (TTE) revealed a left ventricular ejection fraction (LVEF) of 40–45%, with akinesis of the basal-mid inferior segments. These findings led to a coronary angiogram that showed a very large sized, dominant RCA supplying the entire lateral wall; normal left main and left anterior descending (LAD) arteries; and a congenitally absent left circumflex artery (Figure 2). A subsequent cardiac 64 multislice CT scan confirmed the absence of the LCX and showed an RCA with a large posterolateral artery that supplied the entire lateral wall. The LAD was normal (Figure 3). Given the unexplained regional wall motion abnormality, a cardiac MRI was performed which revealed viable myocardium with no areas of late gadolinium enhancement. The calculated LVEF was 40% on the cardiac MRI; the patient was initiated on metoprolol for his frequent PVCs, appropriate therapy for his systolic dysfunction, and discharged home with primary care and cardiology follow-up. On a subsequent Holter monitor, he continued to have occasional PVCs but no ventricular arrhythmias were detected.

## 4. Literature Review

To the best of our knowledge, 20 cases of absent left circumflex artery have been described in the literature that met our inclusion criteria. Our case is the first to be diagnosed due to workup of occasional PVCs but shared some similarities with previously published reports.

Table 1 summarizes the baseline demographics, comorbidities, presentation findings, and diagnostic modalities for all patients. There were 15 male and 8 female patients with a mean ± SD age of 53 ± 10 years. One out of every three patients did not have any cardiovascular comorbidities, whereas the others had known cardiovascular comorbidities. Of these, the most prevalent were systemic hypertension and a history of tobacco use.

The most common presenting symptom that led to the diagnosis of congenitally absent circumflex artery was chest pain. Chest pain on exertion was documented in 59% of the cases; however, these patients did not have any angiographic evidence of coronary artery disease. The etiology of chest pain in these patients is not clearly understood. One of the explanations offered in literature is a steal phenomenon during exertion, where there may be transient ischemia in territories fed by the RCA and LAD due to the diversion of blood flow to anatomical regions usually fed by the left circumflex artery [3].

Uniquely our patient had T wave inversions in the inferior leads and occasional PVCs which led to an ECHO that showed reduced ejection fraction and distinct regional wall motion abnormalities in the territory supplied by the LCX or RCA. This led to the angiogram and the diagnosis of an absent LCX artery. Echocardiograms were reported in 11/21 (52.4%) patients in the review. Apart from our patient, the other 2 patients who had reduced ejection fraction with regional wall motion abnormalities were those with underlying coronary artery disease. The exact etiology of our patients reduced ejection fraction, which was confirmed on the cardiac MRI, was unclear given that he had viable myocardium and no underlying coronary artery disease. Premature ventricular contractions (PVCs) have not been reported as an associated finding in patients with left circumflex anomaly; however, our patient continues to have them despite beta blocker therapy. It is unclear if the PVCs are related to his underlying coronary anomaly.

Percutaneous coronary angiography and computed tomography (CT) of the coronary arteries were the two main diagnostic modalities reported. Like the present case, 12 other patients were diagnosed with a coronary angiography, 3 with cardiac CT and 5 using a combination of coronary angiography and an aortogram. Coronary angiography is the preferred imaging technique for coronary artery evaluation; however, multislice 3D coronary CT has proven to be equally beneficial given its noninvasive nature and three-dimensional view. A combination of both imaging methods obviously gives a more robust assessment of coronary arteries and their neighboring structures [13].

Patients with a congenitally absent LCX usually have a superdominant RCA as an anatomical compensation for blood supply to areas of the heart usually supplied by the LCX. This was evident in our case and was reported in 90.4% of the entire cohort. In addition to the absent LCX, other concomitant congenital abnormalities described so far are (i) atretic mid LAD originating from the sinus of Valsalva, (ii) dual LAD, (iii) LAD originating from the right coronary cusps, and (iv) nonexisting left subclavian artery [10, 15, 19].

The absence of a LCX is usually regarded as a benign condition that does not require lesion specific intervention [12]. Rather, management per guideline directed medical therapy is recommended. None of the 21 cases reviewed reported any negative outcome associated with the absent LCX. Of the entire cohort, 4 (19%) patients were diagnosed with acute myocardial infarction (AMI) with clinically significant stenotic lesions in the remaining blood vessels on angiogram. These patients, however, had significant comorbidities that predisposed them to atherosclerotic coronary artery disease.

## 5. Conclusion

Congenital absence of the left circumflex coronary artery is a rare, relatively benign condition that does not require lesion specific treatment. It presents most commonly with chest pain and may be associated with EKG abnormalities. Definitive diagnosis is usually made by cardiac CTA or coronary angiogram.

## Figures and Tables

**Table 1:** Cases of patients presenting with congenitally absent left circumflex artery.

Ref	Age	sex	Comorbidities	Chest pain	Diagnostic procedure	MI	Echo/nuclear	Associated anomalies of other vessels
Ali et al. [4]	40	M	DM, HTN, smoking	Exertional pain CP	CATH	Yes	Reduced LV function, with inferior and posterior segments were akinetic, anterior, lateral, and septal segments hypokinesis	Large RCA (70% stenosis) and complete occlusion of LAD → CABG, super dominant RCA

Ali et al. [4]	39	M	None	Exertional pain CP	CATH	No	NA	Superdominant RCA

Varela et al. [3]	55	F	None	Nonexertional CP	CATH	No	NA	Superdominant RCA

Oliveira et al. [5]	70	M	Aortic stenosis	Exertional chest pain, syncope	CATH	No	HFPEF 58%, severe AS area < 0.7	Superdominant RCA, anomalous origin of left coronary artery from right coronary sinus

Duan et al. [6]	66	M	Constrictive pericarditis	None	CATH (Pre-op evaluation	No	NA	Superdominant RCA and enlarged LAD branches

Guo and Xu [7]	52	M	HTN, smoking	Nonexertional CP	CATH + CT	Yes	Echo showed severe hypokinesis of the lateral wall, inferior left ventricular wall thinning and akinesis	Superdominant RCA + RCA thrombus, mid portion of LAD stenosis

Quijada-Fumero et al. [8]	51	M	DM, HTN, obesity	None (T wave inversions in V3-v6)	CATH	No	Normal LV, no RWMA	Normal LAD, absent LCX, superdominant RCA

Lin et al. [9]	44	F	None	Exertional CP	CATH	No	Thallium perfusion showed perfusion defects in the septal and inferior walls which normalized in the delayed imaging	Superdominant RCA coronary angiogram

Teunissen et al. [10]	46	M	None	Exertional CP	CATH	No	Normal LV, no RWMA	Mid segment of LAD was atretic originating from left sinus Valsalva, superdominant right coronary artery

Vijayvergiya and Jaswal [11]	40	M	None	Nonexertional CP	CATH	No	Normal LV, no RWMA	Superdominant RCA, LAD originated from the right coronary cusps

Hongsakul and Suwannanon [12]	52	M	HTN, smoking	Exertional CP	CT		Stress test, inconclusive	Superdominant RCA

Majid et al. [13]	55	F	HTN	Nonexertional CP	CT	No	NA	Superdominant RCA

Hong et al. [14]	68	M	HLD	Nonexertional CP	CATH then CT	Yes	NA	Superdominant RCA, with acute thrombosis of RCA

Bildirici et al. [15]	67	F	HTN	Exertional CP	CATH, Confirmed with aortography	No	Normal EF (NRWMA)	Dual LAD, superdominant RCA

Yoon et al. [16]	48	M	HTN, chronic alcoholism	Nonexertional CP	CATH	No	LVH with no other abnormality	Superdominant RCA

Baskurt et al. [17]	55	F	None	Nonexertional CP	CATH, Confirmed with aortography and MDCT (multidetector row Computed tomography)	No	Normal LV, no RWMA	Superdominant RCA

Sato et al. [18]	62	M	CAD	Exertional CP	Coronary CT/CATH	No	NA	Superdominant RCA

Harada et al. [19]	65	F	Aortic stenosis	None	CT coronary (Pre-op)	No	NA	Absence of left circumflex and left subclavian

Doven et al. [20]	67	M	HTN, HLD, smoking	Exertional CP	CATH	No	Normal EF, no RWMA	Superdominant RCA

Harada et al. [19]	49	M	HTN, HLD	Exertional CP	CATH	Yes	NA	Complete Left main occlusion, absent LCX → treated with PCI

Our case	46	M	None	None (frequent PVCs and abnormal Echo findings)	CATH	No	Echo-EF of 40–45%, with mild diffuse hypokinesis with RWMA and akinesis in the basal-mid inferior walls	Superdominant RCA

CATH: coronary angiogram, EF: left ventricular ejection fraction, F: female, HTN: hypertension, LAD: left anterior descending artery, LCX: left circumflex artery, HTN: hypertension, M: male, NA: not available, RCA, right coronary artery, RWMA, regional wall motion abnormalities, STEMI: ST segment elevation MI, and CP: chest pain.
